# Relationship between the percentage of body fat and surrogate indices of fatness in male and female Polish active and sedentary students

**DOI:** 10.1186/1880-6805-33-10

**Published:** 2014-05-13

**Authors:** Grażyna Lutoslawska, Marzena Malara, Paweł Tomaszewski, Krzysztof Mazurek, Anna Czajkowska, Anna Kęska, Joanna Tkaczyk

**Affiliations:** 1 Department of Biology and Biochemistry, Józef Piłsudski University of Physical Education, 00-968 Warsaw 45, Box 55, Warsaw, Poland; 2 Department of Statistics, Józef Piłsudski University of Physical Education, Warsaw, Poland; 3 Department of Sport Medicine, Józef Piłsudski University of Physical Education, Warsaw, Poland; 4 Department of Physiology, Józef Piłsudski University of Physical Education, Warsaw, Poland

**Keywords:** Young adults, Physical activity, Body fat percent, Surrogate indices of adiposity

## Abstract

**Background:**

Limited data have indicated that body mass index (BMI), waist circumference (WC), waist to hip ratio (WHR) and waist to height ratio (WHtR) of athletes and young adults provide misleading results concerning body fat content. This study was aimed at the evaluation of the relationship between different surrogate indices of fatness (BMI, WC, WHR, WHtR and body adiposity index (BAI)) with the percentage of body fat in Polish students with respect to their sex and physical activity.

**Methods:**

A total of 272 students volunteered to participate in the study. Of these students, 177 physical education students (90 males and 87 females) were accepted as active (physical activity of 7 to 9 hours/week); and 95 students of other specializations (49 males and 46 females) were accepted as sedentary (physical activity of 1.5 hours/week). Weight, height, waist and hip circumferences were measured, and BMI, WHR, WHtR and BAI were calculated. Body fat percentage was assessed using four skinfold measurements.

**Results:**

Classification of fatness according to the BMI and the percentage of body fat have indicated that BMI overestimates fatness in lean subjects (active men and women, sedentary men), but underestimates body fat in obese subjects (sedentary women). In all groups, BMI, WHR, WHtR and BAI were significantly correlated with the percentage of body fat (with the exception of WHR and hip circumference in active and sedentary women, respectively). However, coefficients of determination not exceeding 50% and Lin’s concordance correlation coefficients lower than 0.9 indicated no relationship between measured and calculated body fat.

**Conclusion:**

The findings in the present study support the concept that irrespective of physical activity and sex none of the calculated indices of fatness are useful in the determination of body fat in young adults. Thus, it seems that easily calculated indices may contribute to distorted body image and unhealthy dietary habits observed in many young adults in Western countries, but also in female athletes.

## Introduction

In studies concerning health risk, body mass index (BMI) expressed as the ratio of weight to squared height and other easily measured indices of fatness, including waist circumference (WC), waist to hip ratio (WHR) and waist to height ratio (WHtR), are used and recommended by the World Health Organization (WHO) and other organizations as reliable in the assessment of body fatness [1-3].

On the other hand, there are also data indicating that BMI provides misleading results concerning body fat content in different ethnic groups [4,5]. The discrepancy between BMI and percentage of body fat has also been identified in the Caucasian population indicating that BMI ≥30 misses more than half of persons with excess fat [6-8]. Additionally, it has been suggested that WC, WHR and WHtR are specific for ethnicity, and consequently it has been proposed that country-specific cutoff values of surrogate indices of fatness need to be established [9-11].

Similar doubts concerning BMI reliability have been underlined in studies with athletes participating in different sports. Jacobson *et al*. [12] noted that in body builders BMI weakly correlates with the percentage of body fat. Similarly, Mazic *et al*. [13] demonstrated that 28% of basketball players, despite low body fat, were classified as overweight due to BMI higher than 25. Similarly, Garrido-Chamorro *et al*. [14] found that BMI is a poor index of body fatness in athletes representing different sports, since subjects with a low percentage of body fat presented BMI values up to 33. Furthermore, Esco *et al*. [15] found that BMI-based equations for predicting percent of body fat in female collegiate athletes are not appropriate for predicting individual body fat. The limitations of BMI as a reliable index of body fat in young adults have also been observed by others [16]. The BMI as a measure of body fat is inappropriate in adolescent athletes due to incorrect classification of lean subjects as overweight or obese [17]. Moreover, Ode *et al*. [18] showed incorrect estimation of body fat using BMI in college-age males and females, irrespective of their physical activity.

According to our best knowledge, there is no data concerning the relationship between measured body fatness and traditional (BMI, WC and WHtR) and recently introduced (body adiposity index (BAI)) indices of adiposity in young adults with respect to sex and physical activity.

Thus, this study was undertaken and aimed to evaluate the relationship between different surrogate indices of fatness (BMI, WC, WHR, WHtR and BAI) with the percentage of body fat in Polish sedentary and active students.

## Methods

### Subjects

The participants were recruited among students of both sexes on the basis of advertisements in student dormitories and by word of mouth. Before participation, all the students were asked about weekly hours of their physical activity. All the subjects were healthy non-smokers, not taking any medication on a regular basis and gave their written consent prior to participation in the study. The study protocol was accepted by the local ethics commission at the Józef Pilsudski University of Physical Education.

In total, 272 students with an age range of 19 to 22 years volunteered to participate in the study. Of these students, 177 were physical education students (90 males and 87 females) and were accepted as physically active. They participated in different forms of physical activity due to their study program (7 to 9 hours/week of swimming, games, martial arts and running) for at least 12 weeks before the study. None of the active participants were engaged in high performance sports. A further 95 students of other specializations (49 males and 46 females) characterized by physical activity not exceeding 1.5 h/week (gymnastics and/or games) were accepted as sedentary.

### Anthropometric measurements

The percentage of body fat was determined from the sum of the thickness of four skinfolds (biceps, triceps, suprailiac and subscapular), measured using a Harpenden Skinfold Caliper (British Indicators, Burgess Hill, UK) and calculated according to Durnin and Womersley [19]. Body fat mass and lean body mass (LBM) were also calculated. The following values of body fat percentage were accepted as normal: 18 to 24% for males and 25 to 31% for females [20].

After all outer clothing and shoes were removed, body weight and height were measured to the nearest 0.1 kg and 0.1 cm, respectively, using standardized equipment. WC was measured to the nearest 0.1 cm at the level of the iliac crest while the subjects were at minimal respiration. Hip circumference (HC) was measured to the nearest 0.1 cm at the level of the maximum extension of the buttocks in a horizontal plane. Both measurements were performed using non-stretchable tape. All measurements were taken on the right side of the body according to the International Society for the Advancement of Kinanthropometry (ISAK) recommendations [21]. Each measurement was repeated twice and in case of discrepancy was repeated for a third time. BMI was calculated from body mass and body height (kg/m^2^), and body fatness of participants was classified according to WHO standards [2]. WHR and WHtR were also calculated. BAI was calculated according to Bergman *et al*. [22] and the following formula:

$$\text{BAI}=\left[\text{Hip}\;\text{circumference}\left(\text{cm}\right)/\text{height}{\left(m\right)}^{1,5}\right]-18.$$

### Statistical analysis

Data distribution was evaluated using the Shapiro-Wilk test. Data comparison was performed using one-way analysis of variance (ANOVA) and Tukey’s post-hoc test. Correlations between variables were evaluated according to Pearson and subsequently coefficients of determination (r^2^ × 100) were calculated. Statistical significance was set at *P* < 0.05. Lin’s concordance correlation coefficient between the percent of body fat and other indices of fatness were calculated, and concordance correlation coefficient values of at least >0.9 were accepted as indicating significant concordance between variables [23]. STATISTICA version 7 (StatSoft, Tulsa, OK, USA) was used to compute the data, and the results were expressed as means and standard deviations for all variables.

## Results

### BMI and the percentage of body fat

Subjects’ anthropometric characteristics are presented in Table 1. The BMI in active and sedentary females was lower than in males of similar level of activity (*P* < 0.001). However, no significant differences in BMI were noted between active and sedentary females. In contrast, BMI in sedentary males was lower than in their active counterparts (*P* < 0.02).

**Table 1:** Anthropometric characteristics of young sedentary and active males and females

**Variable**	**Active subjects**		**Sedentary subjects**	
	**Males (n = 90)**	**Females (n = 87)**	**Males (n = 49)**	**Females (n = 46)**
Age (years)	21.0 ± 1.8	21.0 ± 2.0	22.0 ± 1.0	21.3 ± 1.4
Weight (kg)	78.4 ± 9.6	61.2 ± 6.7^a^	73.6 ± 8.7^c^	60.6 ± 7.8^h^
Height (cm)	182.4 ± 6.7	170.3 ± 1.2^a^	180.9 ± 6.0	167.2 ± 5.5^g^
BMI	23.6 ± 2.4	21.1 ± 1.6^a^	22.5 ± 2.2^d^	21.7 ± 2.8^h^
Fat (%)	14.4 ± 3.9	25.2 ± 3.3^a^	16.7 ± 4.7	30.9 ± 6.5^g,,h^
Fat (kg)	11.5 ± 4.3	15.5 ± 3.1^a^	12.4 ± 4.0	18.8 ± 5.0^g,h^
LBM (kg)	66.9 ± 6.8	45.7 ± 4.5^a^	61.2 ± 7.2^e^	41.8 ± 5.9^g,h^
Waist (cm)	81.9 ± 6.5	71.3 ± 4.2^a^	78.8 ± 5.2^f^	72.0 ± 5.8^h^
Hip (cm)	98.3 ± 5.3	95.9 ± 5.4^b^	94.3 ± 5.2^e^	95.8 ± 8.1
WHR	0.83 ± 0.04	0.74 ± 0.04^a^	0.84 ± 0.04	0.75 ± 0.06^h^
WHtR	0.45 ± 0.03	0.42 ± 0.02^a^	0.44 ± 0.03	0.43 ± 0.04
BAI	21.9 ± 2.5	25.2 ± 2.5^a^	20.8 ± 2.2	26.3 ± 3.9^h^

Data presented as mean ± standard deviation. Statistical significance evaluated using one-way ANOVA and Tukey’s post-hoc test. ^a^*P* < 0.001 versus active males; ^b^*P* < 0.02 versus active males; ^c^*P* < 0.03 versus active males; ^d^*P* < 0.02 versus active males; ^e^*P* < 0.001 versus active males; ^f^*P* < 0.004 versus active males; ^g^*P* < 0.001 versus active females; ^h^*P* < 0.001 versus sedentary males. ANOVA, analysis of variance; BAI, body adiposity index; BMI, body mass index; LBM, lean body mass; WHR, waist to hip ratio; WHtR, waist to height ratio.

The percentage of body fat in both active and sedentary males was lower than in females with matched physical activity (*P* < 0.001). However, the percentage of body fat did not significantly differ in active and sedentary males, but in active females was lower than in their sedentary counterparts (*P* <0.001).

Classification of fatness according to BMI indicated that irrespective of physical activity most of the participants had normal body fat content (Table 2). The highest percentage of overweight individuals was noted among active males (20%) and the lowest percentage (2.2%) was among active females. In sedentary subjects the percentage of overweight individuals did not differ with respect to sex, and was 10.2% in males and 10.9% in females. In contrast, according to the measured percentage of body fat, 83.3% of active males, 65.3% of sedentary males, 40.2% of active females and 8.7% of sedentary females had low body fat. Normal fat percentage was noted in 15.5% of active males, 34.5% of sedentary males, 59.8% of active females and 41.3% of sedentary females. Obesity was noted in 1.1% of active males and 50% of sedentary females (data not shown).

**Table 2:** Fatness of young adults with different physical activity classified according to BMI values

**BMI**	**Active subjects (%)**		**Sedentary subjects (%)**	
	**Males (n = 90)**	**Females (n = 87)**	**Males (n = 49)**	**Females (n = 46)**
<18.5	-	6.9	-	6.5
18.5 to 24.9	77.8	90.8	89.8	82.6
25 to 29.9	20.0	2.2	10.2	10.9
>30	2.2	-	-	-

BMI, body mass index.

### WC, HC, WHR, WHtR and BAI indices of adiposity

In active and sedentary males WC was higher than in females with similar activity level (*P* < 0.001). Additionally, WC in active males was higher than in sedentary males (*P* < 0.004), but did not significantly differ in females regardless of the activity level.

In active males HC was markedly higher versus females with matched activity level and versus sedentary males (*P* < 0.001 and *P* < 0.02, respectively). In contrast, in sedentary subjects no significant sex-related difference in HC was noted.

Sex-related differences in WHR were observed in both active and sedentary subjects with higher values in males in comparison with females (*P* < 0.001), but no significant differences were noted with respect to activity level.

Significant differences in WHtR values were noted between active males and females (*P* < 0.001) with similar values in sedentary subjects of both sexes.

In both sedentary and active subjects BAI was markedly higher in females than in males (*P* < 0.001). However, physical activity did not affect BAI, since it was similar in active and sedentary subjects of both sexes.

### Associations between measured and calculated body fatness

Correlation analysis revealed that most simply calculated indices of adiposity were significantly related to measured body fat (*P* values from 0.02 to 0.001) with the exception of a non-significant relationship between WHR and body fat in physically active females and HC in sedentary females (Table 3).

**Table 3:** Pearson correlation coefficients between the percentage of body fat and surrogate indices of adiposity

**Variable**	**Active subjects**		**Sedentary subjects**	
	**Males (n = 90)**	**Females (n = 87)**	**Males (n = 49)**	**Females (n = 46)**
BMI	0.667 (44.5)^*^	0.490^a^ (24.0)	0.530^a^ (28.1)^*^	0.438^b^ (19.2)
Waist (cm)	0.720 (51.8)	0.558^a^ (31.2)	0.547^a^ (29.9)	0.557^a^ (31.0)
Hip (cm)	0.650 (42.3)	0.529^a^ (28.0)	0.350^c^ (12.2)	0.091 (0.8)
WHR	0.398^b^ (15.9)	0.052 (0.3)	0.460^a^ (21.1)	0.483^a^ (23.4)
WHtR	0.398^b^ (15.9)	0.470^a^ (22.1)	0.694^a^ (48.2)	0.663^a^ (43.9)
BAI	0.553^a^ (30.6)	0.418^a^ (17.5)	0.600 (36.7)	0.359^c^ (12.9)

^*^Coefficients of determination provided in brackets. ^a^*P* < 0.001; ^b^*P* < 0.003; ^c^*P* < 0.02.BAI, body adiposity index; BMI, body mass index; WHR, waist to hip ratio; WHtR, waist to height ratio.

However, the analysis of coefficients of determination (r^2^× 100) suggested a relatively minor effect of body fat variability on calculated indices of body fatness. The highest values were observed for WC in active males and for HC in active females (51.8% and 42.3%, respectively), and for WHtR in sedentary subjects (48.2% and 43.9% in males and females, respectively). Lin’s concordance correlation coefficients between the percentage of body fat and calculated indices of fatness varied from 0.001 to 0.403 (Table 4).

**Table 4:** Lin’s concordance correlation coefficients between the percentage of body fat and other indices of fatness

**Variable**	**Active subjects**		**Sedentary subjects**	
	**Males (n = 90)**	**Females (n = 87)**	**Males (n = 49)**	**Females (n = 46)**
BMI	0.118	0.173	0.181	0.118
Waist (cm)	0.008	0.007	0.007	0.023
Hip (cm)	0.004	0.004	0.002	0.002
WHR	0.001	0.001	0.001	0.001
WHtR	0.001	0.001	0.001	0.001
BAI	0.137	0.402	0.293	0.231

BAI, body adiposity index; BMI, body mass index; WHR, waist to hip ratio; WHtR, waist to height ratio.

## Discussion

In our study, irrespective of activity level, the BMI of males was higher than females. This finding is in agreement with the data of Pasco *et al*. [24] who indicated that in a large sample of both sexes, BMI overestimates body fat in males mostly due to their higher muscularity and bone mass. Similarly, Nevill *et al*. [25] noted that BMI does not differentiate the adiposity in male and female subjects aged 24.8 to 31.7 years, classified according to training status as athletes and controls.

Our data concerning body fat and lean body mass, as well as the percentage of body fat in young males and females are in agreement with well-documented sex-related differences in body composition (for review see Bouchard *et al*. [26]). Moreover, differences in body composition with respect to physical activity of female participants corroborate other data indicating lower body fat and increased lean body mass in active versus sedentary subjects [27].

Surprisingly, no differences were noted in body fat percentage between active and sedentary males. However, it should be stressed that physical activity has been shown to markedly affect regional but not total body fat, and it could not be excluded that this is the case in our male participants [28,29].

Interestingly, our male sedentary subjects were characterized by a tendency to thinness, whereas sedentary females had a tendency to obesity. It should be noted that a similar trend was found in Polish adolescents [30].

Classification of body fat according to BMI and the percentage of body fat further supported misleading results provided by the BMI, which overestimated body fat in lean participants (active males and females and sedentary males) and underestimated body fat in subjects with a high percent of body fat (sedentary females). These findings are in agreement with other data which questioned BMI validity in body fat evaluation especially in active subjects [31].

Our data concerning WC, WHR and WHtR provide evidence that they are not reliable indicesfor the assessment of body fat, especially in female participants. Their values are similar in active versus sedentary females, despite marked differences in the percentage of body fat. In contrast, in active males WC is higher than in sedentary participants, despite similar percentage of body fat, and is accompanied by similar WHR and WHtR values. A questionable validity of WC and WHR in the evaluation of body fat is in agreement with other data [32].

The BAI of females in our study was higher than in males irrespective of activity level and thus showed a similar trend as the percentage of body fat. However, BAI did not reflect the differences in the percentage of body fat between active and sedentary females clearly seen from skinfold measurements. It should be noted that Esco [33] indicated that BAI results in large individual errors when predicting body fat in female athletes and its reliability was also doubtful in population-based studies [34,35].

Despite all these limitations, all surrogate measures of body fat, except for WHR in active females and HC in sedentary ones, were significantly correlated with the percentage of body fat. However, coefficients of determination (r^2^) indicated that they only slightly reflected the variability in body fatness. Furthermore, Lin’s concordance correlation coefficients between the percentage of body fat and calculated indices of fatness were markedly lower than 0.9, suggesting no relationship between variables.

The reason for this is probably due to the marked influence of muscularity on WC and HC, and in consequence on WHR, WHtR and BAI and the effects of height and muscularity on BMI [36,37]. Thus, neither traditional (BMI, WC and WHtR) nor new (BAI) calculated indices of adiposity provide reliable evaluation of body fat in young adults.

Taking into account the above data, it should be emphasized that surrogate indices of fatness are promoted by the massmedia [38]. Furthermore, the drive for thinness in females and the drive for muscularity in both sexes are common in young adults [39,40]. It thus seems feasible that when self-evaluating body composition from simply measured anthropometric parameters, the young populationare at risk of making inappropriate decisions concerning physical activity and/or dietary habits. Moreover, it should be emphasized that similar practices are wellknown in sportsmen causing spurious weight management protocols, which can be especially dangerous for female athletes [41].

In conclusion, the findings in the present study support the concept that irrespective of physical activity and sex none of the calculated indices of fatness are useful in the determination of body fat in young adults. However, our data concern a relatively small group of subjects. Thus, further population-based studies are needed to recognize if this is true for all Polish students. On the other hand, our data seem to be of importance since easily measured surrogate indices may contribute to distorted body image and inappropriate dietary habits observed in many young adults in Western countries, but also in female athletes.

## Abbreviations

ANOVA: Analysis of variance; BAI: Body adiposity index; BMI: Body mass index; HC: Hip circumference; ISAK: International Society for the Advancement of Kinanthropometry; LBM: Lean body mass; r: Correlation coefficient; r^2^: Coefficient of determination; WC: Waist circumference; WHO: World Health Organization; WHR: Waist to hip ratio; WHtR: Waist to height ratio.

## Competing interests

The authors declare that they have no competing interests.

## Authors’ contributions

The final manuscript was read and approved by all authors. GL carried out the design of the study, interpreted the data and revise the manuscript. MM, AK and JT were involved in data collection and checked the manuscript. PT performed statistical analysis, KM and ACz participated in study design and data interpretation.
